# Multifaceted experiments and photothermal simulations based analysis of laser induced graphene and its fibers

**DOI:** 10.1186/s11671-024-03999-6

**Published:** 2024-03-28

**Authors:** Anurag Adiraju, Ammar Al-Hamry, Aditya Jalasutram, Junfei Wang, Olfa Kanoun

**Affiliations:** Chair Measurement and Sensor Technology, Department of Electrical Engineering and Information Technology, Chemnitz University of Technology, 09107 Chemnitz, Germany

**Keywords:** Laser-induced graphene, Carbon fibers, COMSOL, Photothermal, Fibers, Electrochemistry, Spectroscopy

## Abstract

The interaction of CO_2_ laser with polyimide results in the formation of laser-induced graphene (LIG) and other morphological transitions based on laser parameters, such as Laser-induced fibers (LIF) on the surface. However, a fundamental investigation of LIF, its properties and potential have not been explored until now. We aim therefore to provide novel insights into the LIF by characterization of its structural, electrical, electrochemical, and mechanical properties. Four different morphologies were identified depending on the laser parameters and the temperature required for their formation were quantified by FEM model. Minimum temperatures of 1800 K were required to form LIG and around 2600 to 5000 K to form LIF. High heterogeneity of the LIF along thickness due to temperature gradients, and the existence of sheet structures underneath the fibers were identified. Due to the loosely bound nature of fibers, LIF dispersion was prepared by ultrasonication to functionalize the carbon electrode for electrochemical characterization. The modification with LIF on the electrodes enhanced the electrochemical response of the electrode towards standard redox couple which confirmed the conductive nature of the fibers. This work provides a solid basis for the versatile tuning of the behavior and properties of LIF for potential applications.

**Supplementary Information:**

The online version contains supplementary material available at 10.1186/s11671-024-03999-6.

## Introduction

Graphene, a 2D allotrope of carbon consisting of sp^2^ hybridized carbon atoms [1] is regarded as the building block of other graphitic materials like 0D fullerenes, rolled into 1D nanotubes and stacked on top of each other to form 3D graphite [2]. Since its discovery in 2004 by mechanical exfoliation [3], the applications of graphene have been increasing manifold owing to its remarkable properties like high electron mobility [4], transparency [5], high mechanical stiffness [6], and high thermal conductivity [7]. Standard synthesis methods such as mechanical exfoliation [8], chemical synthesis [9], epitaxial growth on silicon carbide [10] and boron nitride [11] substrates, chemical vapor deposition [12], unzipping of carbon nanotubes (CNT) [13] and microwave synthesis [14] have been implemented in the past for producing graphene. However, scalability, reproducibility, use of toxic chemicals, expensive equipment, and requirement of controlled environments are some of the major bottlenecks associated with these methods. Apart from synthesis, inkjet, aerosol, or screen-printing methods for realizing graphene structures or electrodes have been developed, which offer the advantages of low cost and scalability [15, 16]. Nevertheless, process-dependent multistep protocols such as annealing or coating are necessary after printing, which adds to the fabrication complexity [15].

In light of the above shortcomings, an alternative method by laser fabrication of graphene structures was first developed by Lin et al. [17]. A commercial PI film (Kapton), when irradiated with a laser source with sufficient intensity, due to photothermal interaction resulted in a porous graphene film, and its name was coined as ´laser-induced graphene (LIG). Thereafter, LIG has found its way into various applications [18] such as heaters [19], sensors [20–23], fuel cells [24], antennas [25], and supercapacitors [26]. Recently a comparative study of the LIG films formed by UV and CO_2_ laser concluded that CO_2_ laser aids in reaching LIG with good quality and fewer graphene layers [27].

However, the deciding factors on the obtained morphology of LIG are directly related to the laser parameters used that influence the temperatures induced on the surface. Most of the research [27–29] reports on the formation of sheet structures at low powers, followed by breaking into fibrous structures with an increase in power. However, the mechanism of fiber formation and the resulting properties are not well understood. Fibers generally have the advantage of a high aspect ratio, but whether and under what conditions they can be conductive is not clear. Only a few investigations, such as in [28], demonstrated the necessity of low pixel densities for the formation of fibers. It was suggested that high pixel densities lead to the overlap of pulses and subsequently, the breaking of the fibers. Due to very limited investigations on the laser-induced fibers (LIF), there is a lack of knowledge with respect to the formation mechanism of LIF, its properties, and usefulness.

Apart from the type of morphologies, research on the temperature required to realize LIG on the Kapton surface was also conducted. Wang et al. quantified temperatures around 7972 K at 6 W for engraving the surfaces by FEM simulations [30]. Contrastingly, Li et al. [31] quantified engraving temperatures around 817 °C and 1609 °C for 4 and 8 W, respectively. Biswas et al. [32], quantified the temperatures of 1476 K at 0.65 W, and the processes of laser interaction were defined as carbonization at 673 K and above 773 K as graphitization. Similarly, Ruan et al. [33] established two critical temperatures, 858 K for PI degradation and 2073 K as crater formation temperature or ablation temperatures with maximum temperatures reaching 5434 K at 3 W and 20 mm s^−1^ by a stationary beam. Nevertheless, there is a clear lack of consensus on the surface temperatures to induce different morphologies on the surface.

**Fig. 1:**
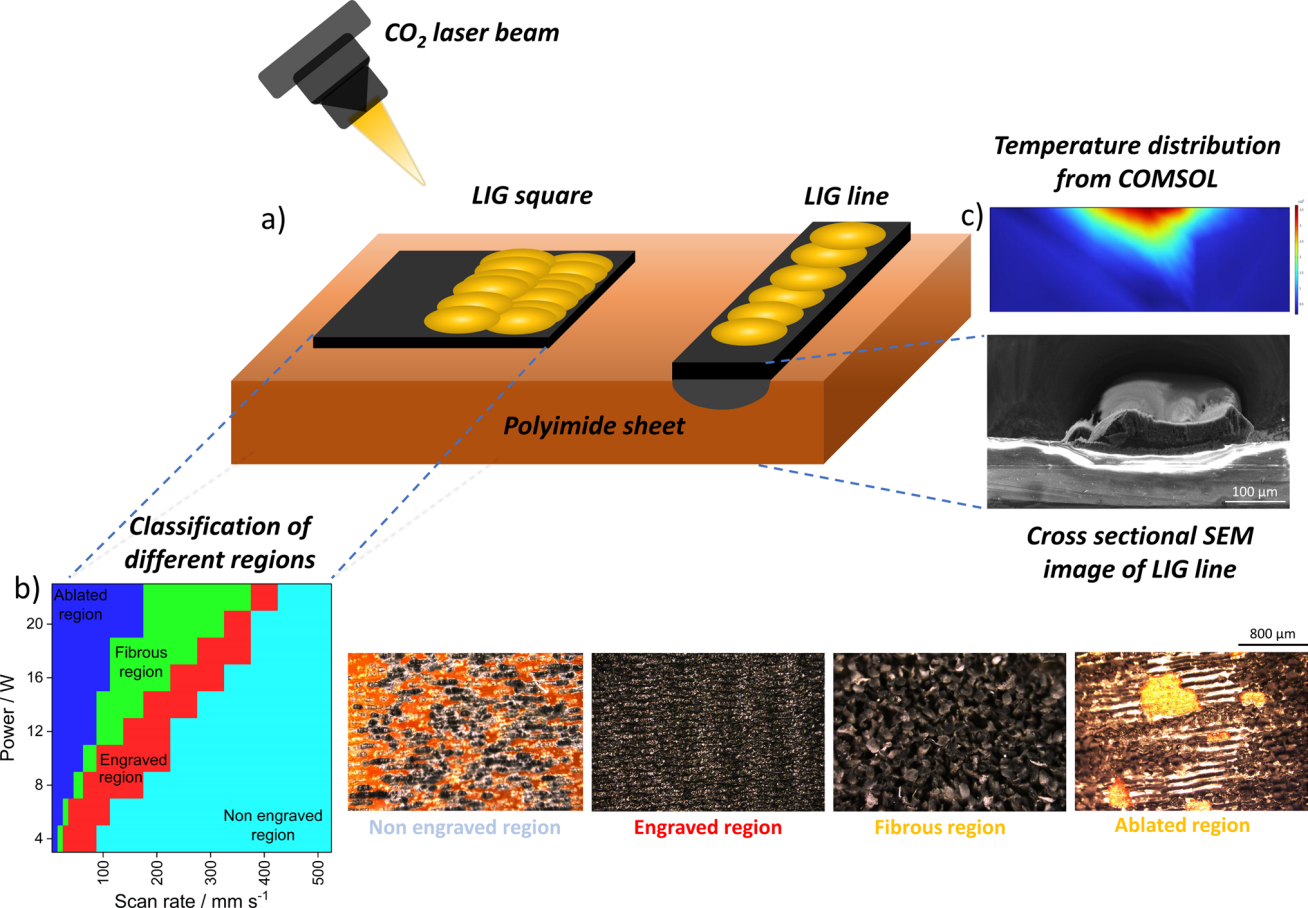
Graphical representation of **a** LIG lines and squares engraved by CO_2_ laser on polyimide sheet, **b** classification of different regions and corresponding optical microscopy images, and **c** cross-sectional SEM images of the line with temperature distribution obtained from simulations

In this regard, the work aims to delve into the systematical elaboration and investigation of LIF. First, the influence of laser parameters on the formation of different morphologies is elaborated. The temperatures required for their formation are evaluated by developing a photothermal model in COMSOL. In addition, the accuracy of the photothermal model is validated through experiments. After the identification of the region for the formation of LIF, through detailed characterization by Raman spectroscopy, optical, scanning electron microscopy (SEM), and Fourier Transform infrared spectroscopy (FTIR), the mechanism for the formation of fibers is elucidated. The properties of the LIF are investigated by different electrical, electrochemical, and mechanical characterizations. Figure 1a shows the graphical abstract depicting the LIG lines and squares that are used for the investigation, Fig. 1b shows the classification of different regions and their optical images, and Fig. 1c shows the cross-sectional image of the lines with corresponding temperature distribution obtained from simulations.

## Materials and methods

### Instrumentation

LIG surfaces on the commercial PI substrate (Kapton from DuPont) was realized by a continuous-wave CO_2_ laser cutting machine (Epilog Mini 24, USA) with a laser spot diameter of approximately 10 µm and wavelength of 10.6 µm. The power can be varied from 0.4 to 40 W and the scan speed from 10 to 500 mm s^−1^. All optical images are obtained through VHX-500 from Keyence. Fourier transform infrared spectroscopy (FTIR) analysis of the surfaces was performed by INVENIO S spectrometer from Bruker, Germany. The measurements were obtained in attenuated total reflectance mode and the influence of the environment was compensated for by the software. Raman measurements were obtained by a Raman plus 532H laser from Metrohm, Germany. The integration time of 60 s and a maximum power of 30 mW was used for spectra acquisition. The scanning electron microscopy (SEM) images were obtained by Nova NanoSEM 200 microscope. Electrochemical measurements were performed by PalmSens 4 potentiostat from PalmSens in a three-electrode configuration.

### Fabrication of LIG structures

Two structures LIG lines and LIG squares are engraved on a Kapton sheet of thickness 110 µm at a fixed resolution of 600 DPI. The Kapton films were cleaned with ethanol and distilled water to remove any impurities prior to engraving. The squares were engraved with fixed dimensions of 5 × 5 mm and the lines with minimum allowable thickness (0.025 mm) and length of 10 mm were selected unless stated otherwise.

LIG squares: To investigate the effect of laser power and scan rate on the obtained surface morphologies, optical images at three different laser powers (4,8, 12 and 20 W) were obtained at different scan rates. Based on the combination of visual inspection and optical microscopy images, four different regions were classified across the powers from 4to 22 W and scan rate from 10 to 500 mm s^−1^. Powers above 22 W were in entirety covered with ablated and fibrous regions and thus were not included in the analysis. SEM images were obtained at five different powers and scan rate of 150 mm s^−1^where fibers are formed (4.8, 8, 10, 12, 14, 16 W). Raman, FTIR analysis was performed on the LIF squares formed at 4.8, 12, and 16 W.

LIG lines: In the case of LIG lines, initially lines were engraved across a certain range of power (8 to 22 W) and scan rate (100 to 500 mm s^−1^) where engraving was achievable. A different range as compared to squares was identified due to the possibility of overlapping of laser beam while engraving the squares. However, LIG lines were engraved by one scan of the laser beam. Further investigation and characterization were performed on LIG lines engraved at 12 W and 150 mm s^−1^. The investigation of cross-sectional images of lines by SEM and subsequently the temperature evaluation by COMSOL was obtained on five different laser powers (8, 10, 12, 14, 16 W) and scan rate of 150 mm s^−1^ similar to LIG squares.

### Analysis of engraved structures

Images of the LIG squares to classify different regions based on the surface morphology were obtained by optical microscope and thorough visual inspection. Further, patterned LIG lines were also imaged by optical microscope, and their line widths were evaluated from the corresponding microscope software. The deformation tests were realized by a lab-made setup consisting of a stepper motor to bend the PI sheet around a roller of 1 cm diameter. All the resistance measurements were calculated by a digital multimeter. The sheet resistance of the squares was measured by a conventional lab-made four-probe measurement setup connected with Keithley 2602 source meter. Electrochemical measurements were carried out in 1 M potassium chloride solution, and the potential range in cyclic voltammetry (CV) was selected from − 0.17 to 0.1 V where no faradic reactions occurred.

### Preparation of LIGF dispersions and its analysis:

For the preparation of LIF dispersion, eight LIF squares at 12 W, 150 mm s^-1^ with area of 9 mm^2^ were engraved on the Kapton. These LIF squares were immersed in a 10 ml isopropanol solution and were ultrasonicated at 20% power for 2 h. The as obtained LIF dispersion was used for modification of the working electrode of carbon electrode. The carbon electrode (ItalSens) consisting of carbon-based working, counter electrode and silver as a reference electrode was purchased from the company PalmSens. The electrodes were tested in a 5mM solution of Ferri-Ferro cyanide prepared in 0.1 M potassium chloride solution by cyclic voltammetry and electrochemical impedance spectroscopy (EIS). For cyclic voltammetry the potential was cycled from − 0.4 to 0.8 V and for the EIS, the frequency range from 0.1 to 15,000 Hz was selected for an AC amplitude of 0.01 V.

### Finite element modelling

The interaction of the laser with PI and the induced temperatures as a function of laser power and scan rate are quantified by finite element simulations using COMSOL Multiphysics. The physics used was 3D Heat transfer with a time-dependent study to account for the photothermal phenomena. A time-variant Gaussian distribution of the laser beam with a spot size of 110 µm was implemented in the model. For accurate modelling of the continuous wave laser, the source was modelled to be moving along a predefined path on the geometry, and the input scan rate of the beam decided its speed. The laser beam was modelled as a Gaussian profile and based on the Lambert- Beers law, volumetric heat source density in 3D in a cartesian coordinate system is given by.

1 $$Q=power \,gp\left(x\right) \,gp\left(y\right) \,\alpha {e}^{-\alpha z}$$

where gp(x) and gp(y) are the Gaussian pulse distribution in the x and y directions, α is the absorption coefficient of Kapton calculated from the FTIR spectra at 10.6 µm, and the exponential term represents the decay of temperature along the thickness of the material. The laser interaction with PI, spatial distribution, and time evolution of temperature are solved in cartesian coordinates by the following partial differential equation in COMSOL:

2 $$\rho {C}_{p}\frac{\partial T}{\partial t}+ \rho {C}_{p}u.\Delta T+\Delta .q=Q+{Q}_{ted}+{Q}_{r}$$

where $\rho$ is the density of Kapton, *u* represents the translational motion, *q* is the conductive heat flux, ${C}_{p}$ is the specific heat capacity at constant pressure, T is the temperature and *Q* is the heat source. *Q*_*ted*_ is thermoelastic damping on the system and ${Q}_{r}$ is the radiative heat source. In the FEM model, the temperature-dependent heat capacity and thermal conductivity of Kapton are given according to the below equations from [34]. Further, the temperature-dependent refractive index is incorporated according to [35]. The emissivity of the Kapton film is given as 0.74, independent of the thickness as reported in [36].

3 $$K\left(T\right)=\left(0.0015\right)+\left(6 {10}^{-4}\right)\left(\frac{T-300}{400}\right)-({10}^{-4}){\left(\frac{T-300}{400}\right)}^{2}$$

4 $${{\text{C}}}_{{\text{p}}}\left({\text{T}}\right)=\left(0.96\right)+\left(1.39\right)\left(\frac{{\text{T}}-300}{400}\right)-(0.43){\left(\frac{{\text{T}}-300}{400}\right)}^{2}$$

where *T* is the temperature and *K(T)*, and *C (T)* are the temperature-dependent thermal conductivity and heat capacity respectively. The initial temperature is set to be considered as ambient temperature (293 K). The geometry consists of a block of dimensions 15,000 × 1000 × 300 µm. A smaller block or strip of dimensions 10,000 × 300 × 110 µm is integrated inside the bigger block for defining the path of the laser beam. The volumetric heat source is given on the top surface, and the remaining sides of the block are thermally insulated. The radiation on the top surface and the thermal insulation are accounted for by implementing necessary boundary conditions. The entire geometry was tetrahedrally meshed with appropriate size by performing mesh convergence studies. The path of the laser beam was meshed extremely fine with tetrahedral elements to resolve the Gaussian beam and for accurate quantification of temperature. The remaining parts of the geometry outside the heat-affected zone were meshed with coarse tetrahedral meshing to minimize the computation time.

## Results and discussion

### Categorization of morphologies and evaluation of temperatures on the surface

**Fig. 2:**
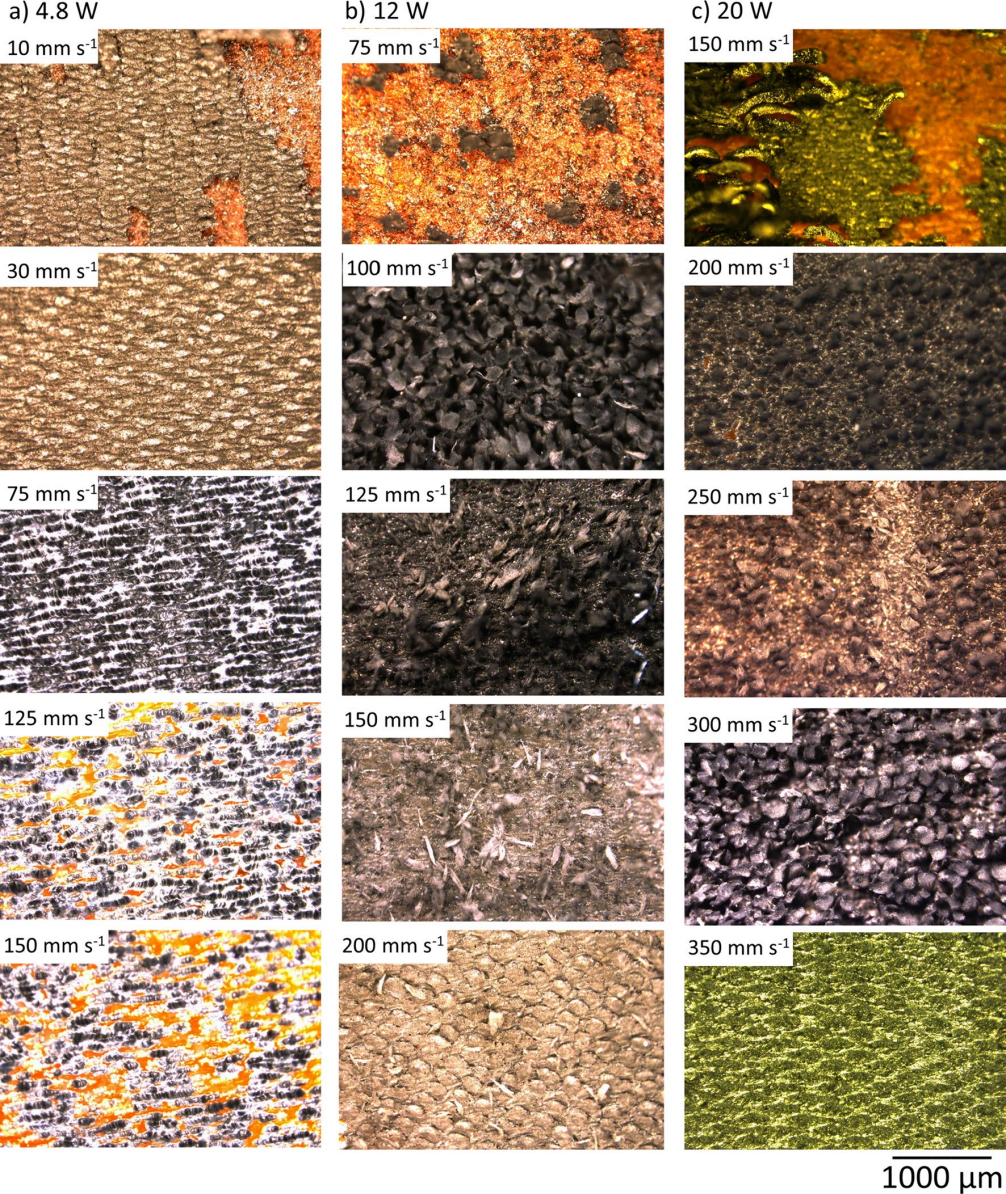
Optical images of squares engraved from top to bottom at **a** 4.8 W with a scan rate of 10, 30, 75, 125, 150 mm s^−1^, **b** 12 W with a scan rate of 75, 100, 125, 150, 200 mm s^−1^ and **c** 20 W at a scan rate of 150, 200, 250, 300, and 350 mm s^−1^

Figure 2 shows the optical microscopy images of engraved squares with laser powers of 4.8, 12, and 20 W at different scan rates arranged for each power column wise to elucidate the influence of laser parameters on engraved LIG.

At low scan rates, the temperature induced is higher, leading to ablation of the surface as observed for 4.8 W at 10 mm s^−1^, 12 W at 75 mm s^−1^ and 20 W at 150 mm s^−1^ in Fig. 2a–c respectively. At 4.8 W and 30 and 75 mm s^−1^ in Fig. 2a, LIG is properly engraved, and further raise in scan rates to 125 and 150 mm s^−1^ does not engrave LIG on the surface as the energy flux induced on the surface are not sufficient to effect any change on Kapton.

In Fig. 2b, at 12 W, the surfaces are ablated at 75 mm s^−1^, fibers are the first morphological change observed at 100 mm s^−1^, and their density reduces as the scan rate rises to 125 and 150 mm s^−1^. At 200 mm s^−1^, the energy flux on the surface is in the range where LIG is formed with very less fibers on the surface. In Fig. 2c, at 20 W, fibers are initially disintegrated at 200 and 250 mm s^−1^ due to high energy induced on the surface. However, high density of fibers was found at 300 mm s^−1^. Upon further increase to 350 mm s^−1^, LIG is formed without any fibers on the surface. Thus, it can be concluded that a certain energy flux is required for the formation of LIF. Any changes in the laser parameters that lead to higher or lower energy flux might disintegrate the fibers or lead to formation of LIG void of fibers respectively.

**Fig. 3:**
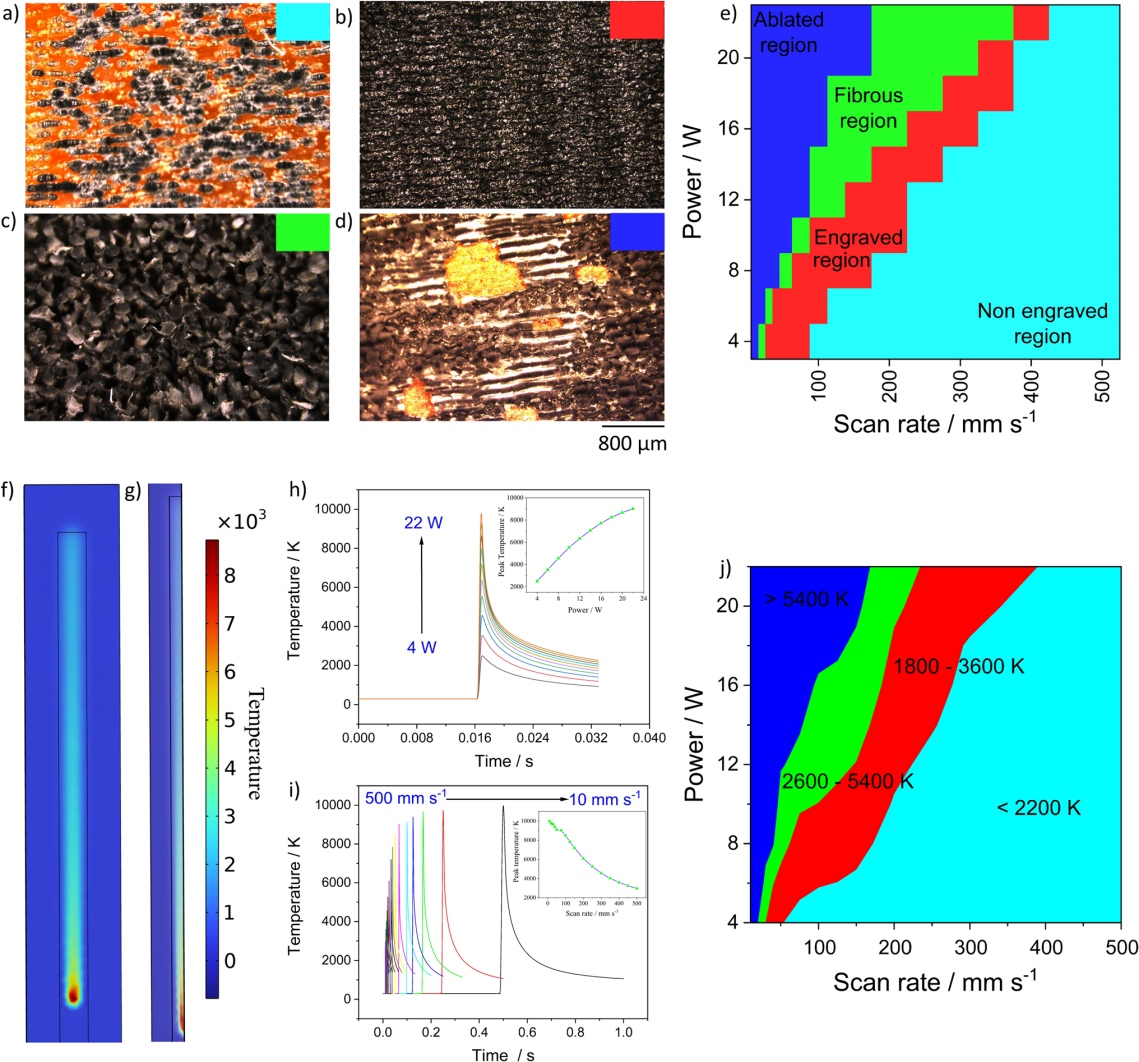
**a**, **b**, **c**, **d** Optical images of not engraved, engraved, fibrous, and ablated surfaces, **e** Heat map plot of the four regions at different powers and scan rates, **f** image of the modeled laser beam on Kapton in COMSOL, **g** Cross-sectional image of the modelled  laser beam with temperature as the scale bar, **h** shows the plot of temperature versus time as a function of power at scan rate of 300 mm ﻿s^−1^ with inset showing the variation of peak temperature w.r.t scan rate **i** depicts the plot of temperature versus time at varying scan rate and constant power of 8 W with inset showing corresponding variation in peak temperature versus the laser power and **j** shows the conotur plot of temperature evaluated for the formation of four different surfaces from COMSOL

In this regard, based on the analysis by optical images, four different regions as seen in Fig. 3a) not engraved, 3b) engraved, 3c) fibrous, and 3d) ablated regions are identified. Herein, the engraved region is the LIG surface with no fibers visible by an optical microscope. However, the existence of fibers, even though in very low density on these surfaces cannot be ignored as seen in Figure S1 in the supplementary information where a small number of fibers protrude out from the LIG surface. Considering the subtle transitions, the classification of the regions was performed by laying down certain standards such as microscopically visible fibers in the first instance to be categorized in the fibrous region. Not engraved and ablated regions convey their usual meaning and are formed when low or high temperatures are induced on the surface respectively.

Figure 3e shows the heatmap plot depicting the distribution of the four regions across powers from 4 to 22 W and scan rate from 10 to 500 mm s^−1^. The most dominant region, mainly prevalent almost in entirety at very low powers is the non-engraved, where sufficient temperatures are not induced to transform the Kapton into graphene. On the other extreme at high powers, the ablated region are mostly prevalent as high temperatures are induced mainly at very low scan rates. In between the two extremities, are two narrow strips representative of fibrous and engraved regions. The fibrous part is the last morphological transformation observed right before the ablation of the surface and is also noticeable in Fig. 3d, wherein fibers are also present on the surface apart from the ablated regions. Similarly, the transition between the engraved and fibrous regions is not obvious, and its boundaries are blurred as the probability of the existence of fibers in the engraved regions cannot be ignored. The engraved region as stated above are the LIG surfaces which are the regions widely used and optimized in literature.

As the process is photothermal and the surface morphologies depend on the laser parameters, it is worthwhile to investigate the induced temperatures on the surface. A FEM model of a traveling laser beam with Gaussian distribution is modelled to evaluate the temperatures induced on the Kapton film, and Fig. 3f shows the 2D image of the traveling laser beam on Kapton. The color scale shows the temperature distribution as the laser beam travels on the Kapton sheet. The image and the temperature values were obtained at 12 W of laser power, scan rate of 150 mm s^−1^ and at time 0.033 s. Figure 3g shows the cross-sectional image of the surface depicting the laser beam distribution along the thickness of the Kapton sheet. Figure 3j depicts the temperatures required for the formation of the four different regions observed previously (Fig. 3e). As can be seen, temperatures less than 2200 K led to partial or no engraving on the surface and temperatures above 5400 K leads to ablation of the surface. Fibrous regions are formed at temperatures ranging from 2600 to 5400 K, and efficient engraving is possible from 1800 to 3600 K. Previous studies concerning the temperatures concluded 2000 to 3000 K as an appropriate range for the formation of LIG structure and any value greater than 3500 K leads to complete disintegration of the material [37]. In our investigation, the temperature range required to observe the four regions has been elaborated and quantified by simulations of a photothermal model. However, slight variations in temperatures are to be expected considering the overlap between the different regions.

The evolution of temperature at a particular position as a function of power at 300 mm s^−1^ and as a function of scan rate at laser power of 8W, was investigated and the findings are shown in Fig. 3h and i respectively. The induced temperatures on the surface at a constant scan rate rises with the increase in the laser power from 4 to 22 W as seen in Fig. 3h. The inset in the figure shows the rise in peak temperature obtained from the maximum of the curves with rise in laser power. Figure 3h shows the temperature profile at a particular position on the strip where the laser beam travels by varying the scan rate from 500 to 10 mm s^−1^. As expected, the temperatures on the surfaces reduces with rise in scan rate and as the duration the laser beam spends at a particular position is lower with rise in scan rate. Inset shows the dependency of peak temperature on the scan rate Nevertheless, such high temperatures are assumed to be slightly overestimated by COMSOL, because the texture and morphology of the engraved LIG could lead to scattering effects that are not modelled, and evaporative flux loss during the phase change is also not accounted [38]. However, the estimated temperatures by COMSOL and the analysis of the variation in surface morphology indeed provides a fundamental understanding of the complex interaction between infrared laser and Kapton film.

### Formation mechanism of LIF and its physical, electrical and electrochemical properties

**Fig. 4:**
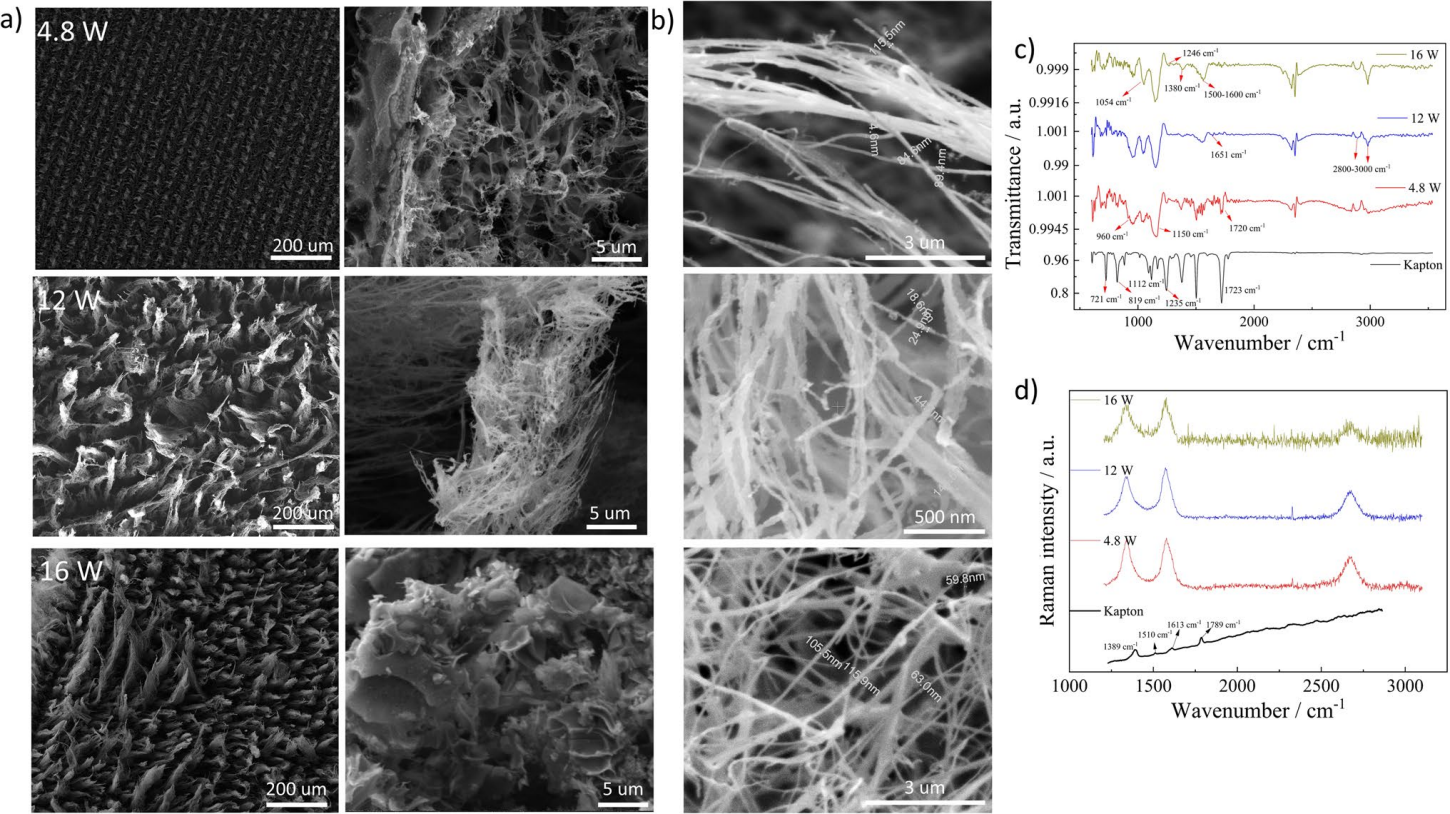
**a** SEM images of the squares at a laser power of 4.8, 12, and 16 W, **b** shows the magnified SEM images of fibers obtained at three different locations on the squares engraved at 12W , **c** and **d** shows the FTIR and Raman spectra of squares engraved at 4.8, 12, and 16 W, respectively

A detailed investigation of the surface morphology and corresponding nanostructures formed at different powers by SEM images at 4.8, 12, and 16 W is shown in Fig. 4a. In supplementary information, the SEM images for 8, 10, and 14 W are provided in Figure S2.

At 4.8 W, the porous morphology is quite visible, along with fibers distributed on the center of the laser path where the temperatures induced are the highest. The magnified image to the right shows amorphous viel and web-like structures along the laser path. With the increase in laser power, abrupt changes to highly dense vertically aligned fibrous forests are visible on the surface at 12 W and is in accordance with previous findings [33]. The dimensions of these fibers were obtained from three surfaces engraved with the same laser parameters and the corresponding SEM images can be seen in Fig. 4b. Generally, a high aspect ratio could be identified with dimensions of individual fibers ranging from 20 to 150 nm. However, further analysis and more statistical information obtained from a greater number of positions on the surface and also the information about the lateral dimension of the fibers are required to confirm and quantify the aspect ratio of the fibers. From the SEM image of the surface at 16 W seen in Fig. 4a, the length of the fibers at certain locations are considerably higher compared to others. The larger length of fibers could possibly lead to bending of these structures and there by exposing the underlying surface as seen in Figure S2 in the supplementary information. Further similar regions were identified for surfaces engraved at 14 W. The magnified image of the surface at 16 W to the right obtained from a different location shows a unique morphology that resembles bulk polymer structure with short and randomly oriented LIFs.

To elucidate the mechanism, the interaction of laser beam with Kapton sheets that leads to breaking of bonds and outgassing of small molecules such as CO_2_, H_2_, and CO leading to the formation of aromatic rings [39–41]. The fibrous forests formed at all the powers above 4.8 W could be related to the high temperatures induced that leads to swelling and, eventually, localized blasts occurring on the surface [37]. The unique solid morphology with randomly oriented fibers identified at 16 W was not reported before, as it has been widely believed that the stage after fiber formation is its breakage into carbon nanodroplets [28] before the ablation of the surface.

Figure 4c shows the FTIR spectra of Kapton and LIG engraved at 4.8, 12, and 16 W. The standard bands for the Kapton film namely C–N–C out of plane bending at 721 cm^−1^, out of plane bending vibrations of aromatic rings at 815 cm^−1^, imide C–N–C transverse stretching at 1112 cm^−1^, non-cyclic C–O–C stretching at 1235 cm^−1^, and C=O out of plane vibrations at 1723 cm^−1^ were obtained [42]. With respect to LIG, starting from the high wavenumbers, the bands present between 2800 to 3000 cm^−1^ correspond to symmetric and asymmetric stretching of saturated methyl groups on the surface [43]. Moving further, the band at around 2350 cm^−1^ is related to the asymmetric stretching of CO_2_ due to the surrounding environment. Specifically, the band at around 1720 cm^−1^ ascribed to the stretching of the carbonyl groups is present only for surfaces engraved at 4.8 W. On the other hand, the band at 1560 cm^−1^ is observed for 12 and 16 W, which corresponds to the *sp*^2^ network [44] on the surface. Bands at 1380 and 1246 cm^−1^ are observed at 4.8 and 16W, corresponding to the C=O stretching and C–O–C stretching of the epoxide group [45], which are absent for 12 W. The two intense bands at 1040 and 1150 cm^−1^ exist for the three powers, indicating the C–O stretching vibrations [46]. Overall, by FTIR analysis, the existence of oxygen functionalities on the surface of LIG is confirmed irrespective of the laser power. However, similar functional groups such as C = O and C-O were identified for 4.8 and 16 W, due to the fact that at 16 W, the fibers are uprooted as seen in SEM image in Fig. 4a which exposes the underlying surface. At 12 W, where fibers are particularly high on surface, the dominance of C–O groups in the spectra is observed.

Raman spectroscopy is a versatile tool to analyze different carbon materials and was used to analyze the LIG squares formed at 4.8, 12, and 16 W. The Raman spectra of Kapton shown in Fig. 4d, consists of peaks at 1389 cm^−1^, 1613 cm^−1^, 1789 cm^−1^ that are related to the C–N stretching vibration of the imide system, C=C stretching vibrations and stretching vibrations of C=O respectively. Figure 4c shows the normalized Raman spectra of the three LIG surfaces after performing the baseline correction. The disorder-related peak at around 1345 cm^−1^ (D peak), E_2_g mode that characterizes the graphene at 1580 cm^−1^ (G peak), and a typical overtone of D peak centered around 2670 cm^−1^ (2D peak) were observed which are typical for carbon related materials [47]. The ratio of intensities of the D band to the G band provides an estimate of the defects and extent of disorderness on the surface and its values were 0.94, 0.78, and 0.86 for 4.8, 12 and 16 W, respectively. The high *I*_d_/*I*_g_ ratio obtained for the surface engraved at 4.8 W indicates that the temperatures are sufficient to induce more defects rather than graphitize the surface. As the power rises to 12 W, sufficient temperatures are induced to invoke more graphitization of the sample compared to defects [48]. However, the rise in the ratio at 16 W is linked to the partial ablation of the surface that exposes the underlying defective regions, which was confirmed by FTIR analysis.

**Fig. 5:**
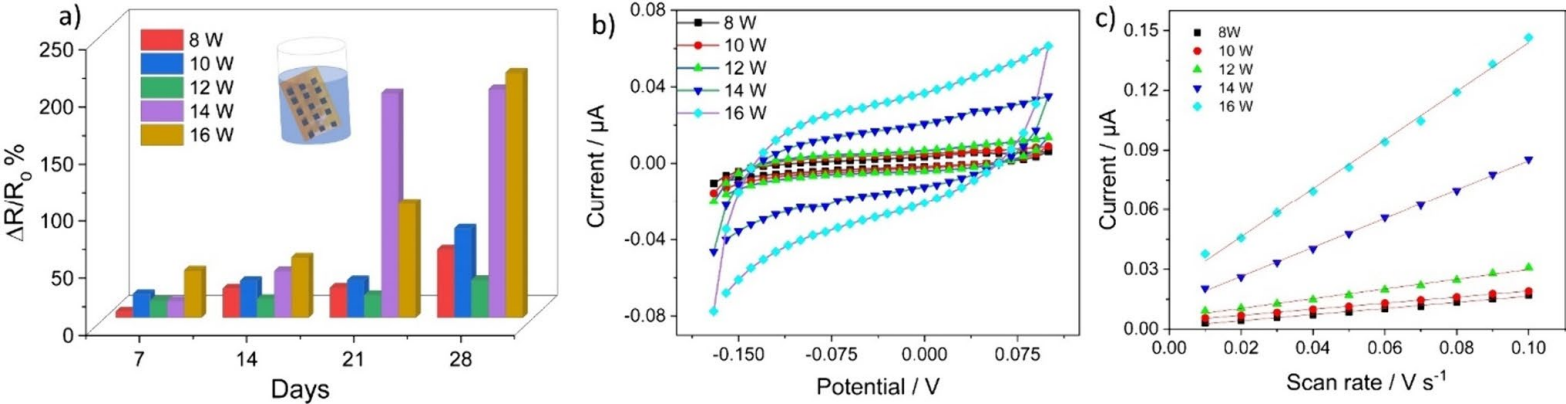
**a** Depicts the relative change in resistance as a function of the number of days of immersing in water, **b** shows CV curves in 1 M potassium chloride solution recorded for different powers and **c** shows the linear plot of currents obtained at exactly the center of the CV curve versus scan rate

To investigate the feasibility of LIF surfaces towards applications involving liquid media, squares (5 × 5 mm^2^) of LIF engraved at different powers were immersed in distilled water and its sheet resistance was evaluated for a period of one month as seen in Fig. 5a and its inset. The relative changes in the resistance of the surfaces increase with the number of days for all the powers which infers that the fibers are loosely bound to the surface. A significant change in the resistance was observed for 14 and 16 W which could be related to the high density of fibers on the surface and also concludes that fibers are more loosely bound and could be removed easily which is confirmed through SEM images of the surface at 16 W. The capacitance offered by the LIF surfaces at different powers was investigated through cyclic voltammetry curves collected from a scan rate of 0.01 to 0.1 V s^−1^ in 0.1 M potassium chloride solution. The curves in Fig. 5b show a pseudo rectangular behavior that is indicative of good capacitive surfaces [49]. Figure 5c shows the current versus the scan rate obtained exactly at the center of the CV curve from Fig. 5b at different scan rates. The capacitance values calculated from the slope of Fig. 5c at different powers, increases from 2.172 at 8 W to 2.185, 3.471, 10.4, and 17.42 µF cm^−2^ for 10, 12, 14, and 16 W, respectively. The increase could be related to the rise in the density of the fibers on surface with rise in temperatures.

### Electrical and microscopic characterization of LIG and LIF lines and evaluation of temperature distribution along the thickness

**Fig. 6:**
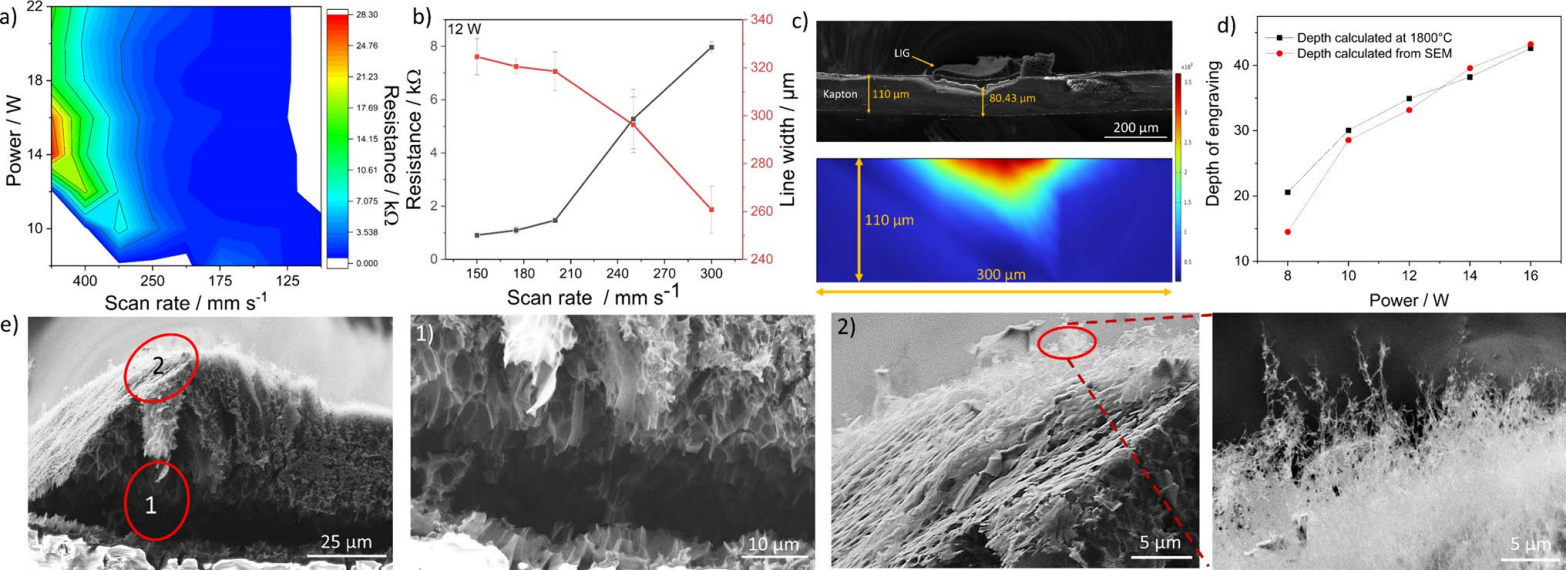
**a** Contour plot of resistance offered by engraved lines at different powers and scan rates, **b** shows the plot of the variation of resistance and line width as a function of scan rate of line engraved at 12 W, 150 mm s^−1^, **c** cross-sectional SEM image of line engraved at 12 W, 150 mm s^−1^ depicting the engraved depth and its corresponding temperature distribution along the depth modelled in COMSOL, **d** Comparison plot of engraved depth evaluated from COMSOL at 1800 °C and calculated from SEM images for different powers, and **e** shows the cross sectional SEM images of the LIGF at 12 W, 150 mm s^−1^ consisting of sheet structures underneath (labelled as 1) and fibrous structures at the top (labelled as 2 including its magnified image)

The effect of laser parameters on the conductivity was investigated by engraving lines by a single pass of the laser beam on Kapton. Figure 6a shows the contour plot of quantified resistances across the possible range of power and scan rate measured in triplicate by digital multimeter, and measured line width using an optical microscope. The regions in white in the contour plot represent either non-engraved or ablated lines. To understand the behavior, Fig. 6b shows the variation in resistance and line width as a function of scan rate at 12W. The resistance increases with the rise in scan rate as enough temperatures are not induced to graphitize the samples. On the contrary, the line width reduces with an increase in scan rate as the heat-affected zone is lower due to the shorter duration of the beam at a particular location.

To investigate the depth of engraving and the corresponding temperature profiles along the thickness, cross sectional images of the lines were obtained at 8, 10, 12, 14, and 16 W at 150 mm s^−1^. Figure 6c shows the cross-sectional image of the LIF line engraved at 12 W. The SEM images for the remaining powers are provided in the supplementary information in Figure S3. The depth of engraving increases from 14.5 to 43.23 µm as the power rises from 8 to 16 W as seen from Fig. 6d. The observations are obvious as the induced temperatures are more at high powers which leads to deeper gradients of temperatures along the thickness. Figure 6c also shows the temperature profile obtained from simulations along the depth at 12 W and 1800 °C was considered as the minimum temperature required for engraving as quantified from Fig. 3i. The obtained values of the depth of engraving at this temperature calculated from the cross-sectional image from the simulations were almost in the similar range to that obtained from SEM images, as can be seen in Fig. 6d. In addition, to cross validate the results, the values of the temperatures were quantified exactly at the experimentally measured values of the depth. The values quantified were 2073.9, 1925.1, 2122.3, 2075.1, and 1813.8 K for laser powers from 8 to 16 W, respectively. All the temperature values lie almost near the vicinity of 2000 °C. Also, it was identified from Fig. 3i that temperature below 2200 K leads to no engraving on the surface and all the values were below 2200 K. These results suggest the efficacy of the developed model and confirm that temperatures of around 1800 °C are necessary to induce modification on the Kapton surface. However, one of the important points to highlight is that the temperature profiles were derived at the moment the laser beam tip touches the prescribed location, and the profile after the laser beam is entirely in the prescribed location is shown in Figure S4 in the supplementary information. Apart from the underlying surface and depth of engraving, the 3-dimensional (3D) surface morphology of LIGF was explored through magnified images of Fig. 6c and are shown in Fig. 6e. The leftmost image in Fig. 6e illustrates the 3D morphology of the LIGF surface. The highlighted positions in the image named as 1 and 2 show the presence of sheet structures and fibrous morphology. The images of the highlighted positions reveal an interesting fact that sheet and fibrous structures coexist on LIGF surface wherein the sheet structures are present underneath the fibers and also infers that not all the sheet structures are disintegrated to form fibers.

### Mechanical exfoliation, deformation analysis and electrochemical characterization of LIGF dispersions

As the fibers are superficially bound to the LIG surface, the influence of these fibers on the electrical and structural properties of the surfaces was investigated. In this regard, LIF lines at 12 W, 150 mm s^−1^ were engraved on the surface and mechanical exfoliation of the surfaces by scotch tape was performed. After every exfoliation step, electrical resistance of the line was evaluated, and the corresponding Raman spectra of the surface was acquired. Both the measurements were taken in triplicate, and as the method of exfoliation was manual, best efforts was made to ensure that same force is applied for each exfoliation. Unlike lines with minimum possible thickness that is possible to engrave with single pass of laser beam for the analysis in Fig. 6, herein lines of thickness 1mm were engraved such that the surface is more densely covered with fibers, this provides the possibility to perform more mechanical exfoliations and thereby allowing for the detailed analysis of the LIF. To illustrate the difference of line engraved in a single pass and in multiple passes, SEM image of lines at 12 W and 150 mm s^−1^, but with different thickness are shown in Figure S6 in supplementary information.

**Fig. 7:**
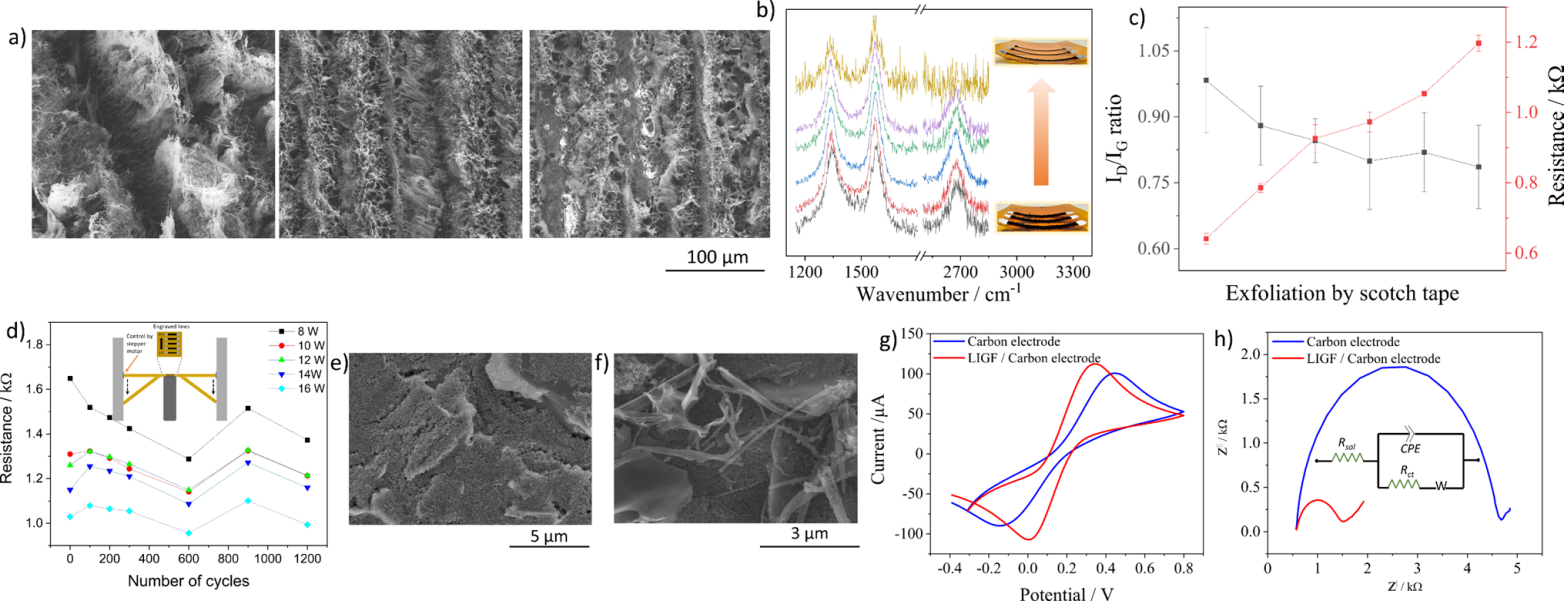
**a** SEM images showing the LIF line of thickness 1 mm engraved at 12 W, 150 mm s^−1^ with center image after the 3rd exfoliation and the right image shows the surface after 6th exfoliation, **b** shows the corresponding evolution of Raman spectra after every exfoliation with inset showing optical images of the surface, **c** depicts the evolution of Id/Ig ratio and electrical resistance of lines measured in triplicate as a function of number of exfoliations, **d** changes in the resistance during the deformation tests at 8,10,12,14, and 16 W and inset shows the schematic of the setup used for the experiments, **e**, **f** shows the SEM images of bare carbon electrode and LIF modified carbon electrode respectively, and **g**, **h** shows the cyclic voltammetry and EIS curves obtained for both the electrodes in standard redox probe solution respectively and inset in h shows the corresponding equivalent circuit used for fitting

Figure 7a, starting from the left shows the LIF line without any exfoliation which consists of significant number of fibers on the surface, followed by the surface after exfoliating for three times (center image) wherein the fibers were almost exfoliated from the surface. The image to the right shows the surface after 6 exfoliations wherein underlying Kapton was visible at certain regions on the line.

Figure 7b shows the evolution of Raman spectra and 7c shows the evaluated *I*_*d*_/*I*_*g*_ ratio after normalization and peak fitting and electrical resistance of the lines after every exfoliation step. As can be seen from Fig. 7c, the *I*_*d*_/*I*_*g*_ ratio decreased from 0.98 for the non-exfoliated sample to 0.79 after the exfoliation for the fourth time, as seen in Fig. 6d. Upon further exfoliation, a slight increase in the ratio was observed. Further, an upward trend in resistance values was observed after removing the fibers initially within the first three exfoliation steps and the LIG surface as well for the next steps. The observed behavior could be related to the heterogeneous nature of the LIG along its thickness or the inner surfaces. On the outer surfaces, where the temperatures induced are the highest, fibers are formed, which could possibly have led to higher conductivity. After successive exfoliations, in the inner layers where moderate temperatures are induced, graphitization is a more dominant mechanism than the defects. At the lowest possible engraved surface, the temperatures induced lead to formation of defects as more dominant than graphitization of the Kapton. One important observation from the Raman spectra is the disappearance of the 2D peak after a series of exfoliations, which could be attributed to the lack of order in the stacking of graphene layers. To conclude, at low temperatures, in the inner layers, amorphous carbon is formed and by moving closer to the surface the temperatures induced are higher that leads to structural ordering in the form of fibers.

The mechanical capability and the durability of LIF surfaces was investigated by bending the Kapton substrates with lines engraved at 8, 10, 12, 14 and 16 W at center of the sheet as shown in the inset of Fig. 7d. The changes in resistance of lines engraved at different powers were quantified as a function of the number of cycles wherein one cycle is equivalent to deformation until the Kapton does not change its behavior and coming back to the original position. The extent of deformation to be performed was calibrated in prior by testing the maximum range up to which Kapton could be bent without losing its elastic behavior. Except for 8 W, an increase in resistance after the first 100 cycles of bending for all the powers was observed. This is related to the removal of superficially bound fibers that are loosely in contact with the surface at all. From thereafter, the resistances reduced with an increase in the number of cycles up to 600 after which an increase in resistance for lines at all the powers was observed. Nevertheless, the measurements conclude minute changes in resistance values after 1200 cycles of bending but are not sufficient to prove the feasibility of fibers for strain measurements.

As it was concluded that fibers are loosely bound to the surface from previous investigations, the fibers were extracted from the surface as a dispersion in isopropanol and the methodology implemented is elaborated in material and methods section. The as prepared dispersion was modified on the working electrode of screen-printed carbon electrode and Fig. 7e and f shows the SEM images of the bare carbon electrode and LIF modified carbon electrode. As can be seen, the LIF modified surfaces consists of several fibrous structures although of varied dimensions on the surfaces. Further bunch of fibers or entangled structures were also identified on the surface. The diversity in the fibers could be attributed to either the ultra-sonication procedure itself or the possibility that these fibers were already of such nature on the LIF surface. Nevertheless, the performance of these fiber modified carbon electrode was investigated by electrochemical measurements using cyclic voltammetry and electrochemical impedance spectroscopy in a standard redox probe solution as can be seen in Fig. 7g and h respectively. From the cyclic voltammetry curves, it is observed that the modification of fibers enhanced the current response of the electrode from 64.75 to 109.6 µA and also reduced the peak potential difference from 0.54 to 0.32 V which indicates faster and higher electron transfer between electrolyte and the electrode due to the conductive nature of the fibers. Figure 7h shows the standard Nyquist plot of both electrodes wherein the diameter of the semi-circle region indicates the charge transfer resistance offered by the electrode. To evaluate the charge transfer resistance, the spectra was fit by Randles equivalent circuit shown in the inset of the Fig. 7h where in starting from high frequencies to the left, R_sol_ is the solution resistance, R_ct_ is the charge transfer resistance, CPE is the constant phase element and W is the Warburg element at low frequency region. Based on the fitting, the obtained values of charge transfer resistance were 3993 and 914.7 for bare carbon and LIGF modified carbon electrode respectively. The obtained values confirm the modification of carbon electrode by LIF enhances the conductivity and thereby the charge transfer capability of the electrode.

## Conclusion

In this paper, a deep investigation of the morphological, structural, mechanical, optical, and electrical properties of LIF was reported for the first time. The photothermal interaction between CO_2_ laser and polyimide leads to the carbonization of the substrate and based on the selected choice of laser parameters, fibers are formed on the surface. The LIFs are the penultimate morphological change observed right before ablation. Four different regions were classified across the entire parameter range and for the first time, to the best of our knowledge, temperatures required to obtain these surfaces were quantified by FEM simulations by modelling a travelling laser beam with a Gaussian profile. FTIR and Raman spectroscopy of the fibers revealed the presence of functional groups and defects, respectively. The capacitive behavior of the LIF surfaces at different surfaces evaluated by cyclic voltammetry increases with the rise in power indicating the increase in fibers density on the surface. In addition, Raman spectroscopy after every exfoliation by a scotch tape of engraved lines revealed highly heterogeneous layers of LIG due to the temperature gradient along the surface. The analysis of LIF surfaces by immersion and mechanical exfoliation experiments revealed loosely bound nature of these fibers and also indicated a rise in resistance after removing the fibers with every exfoliation. The conductive nature of the fibers was observed by increase in current response and decrease in charge transfer resistance after modification of the bare carbon electrode with LIF dispersion. Although the LIF are superficially bound to the surface, which limits their applications in several areas, their properties cannot be ignored. The outcomes of this research would provide novel fundamental insights into different morphologies of LIG, nature, and properties of the LIFs, the temperatures required for their formation. Further, the research would act as a platform for initiating future research that involves potential use of LIF for several applications.

### Supplementary Information

Supplementary file.

## Data Availability

The data presented in this research will be provided by the corresponding author upon reasonable request.
