# Supplementary material for: Multifaceted experiments and photothermal simulations based analysis of laser induced graphene and its fibers
Source: Discov Nano. 2024 Mar 28;19(1):59. doi: 10.1186/s11671-024-03999-6 (PMC10978564; doi:10.1186/s11671-024-03999-6)
Supplement: Supplementary file 1 — Supplementary file. [file 11671_2024_3999_MOESM1_ESM.docx]

Supplementary information

Multifaceted experiments and photothermal simulations based analysis of laser induced graphene and its fibers.

**Anurag Adiraju^1*^, Ammar Al-Hamry^1^, Aditya Jalasutram^1^, Junfei Wang^1^, Olfa Kanoun^1^**

^1^ Chair Measurement and Sensor Technology, Department of Electrical Engineering and Information Technology, Chemnitz University of Technology, 09107 Chemnitz, Germany

E-mail: adiraju.anurag@etit.tu-chemnitz.de


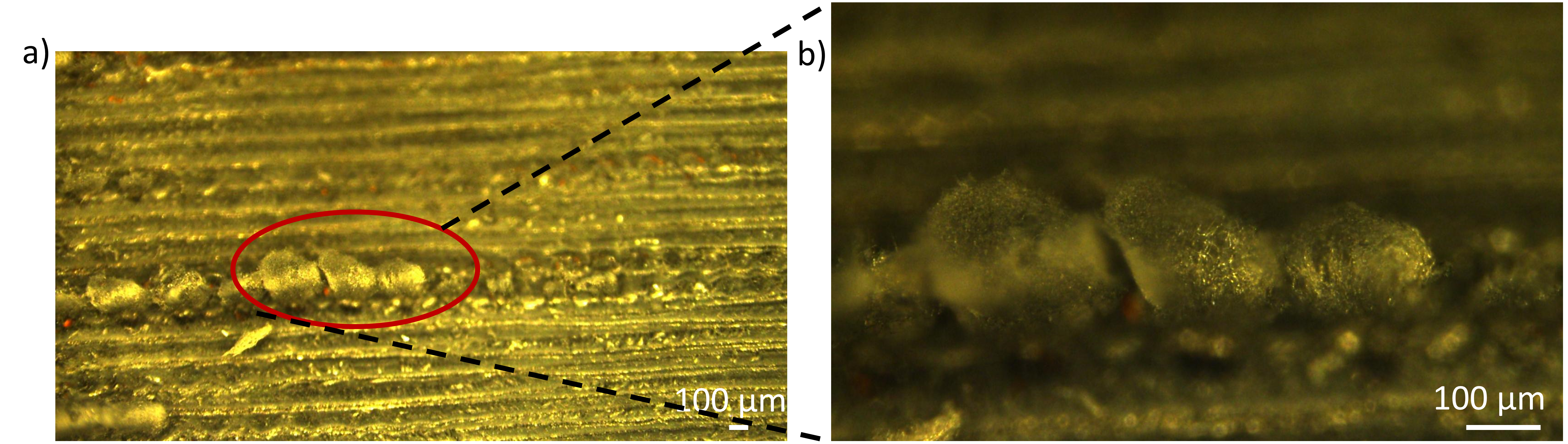


Figure S1: Optical image of a) engraved surface and b) shows the magnified image highlighting the fibers protruding out from surface


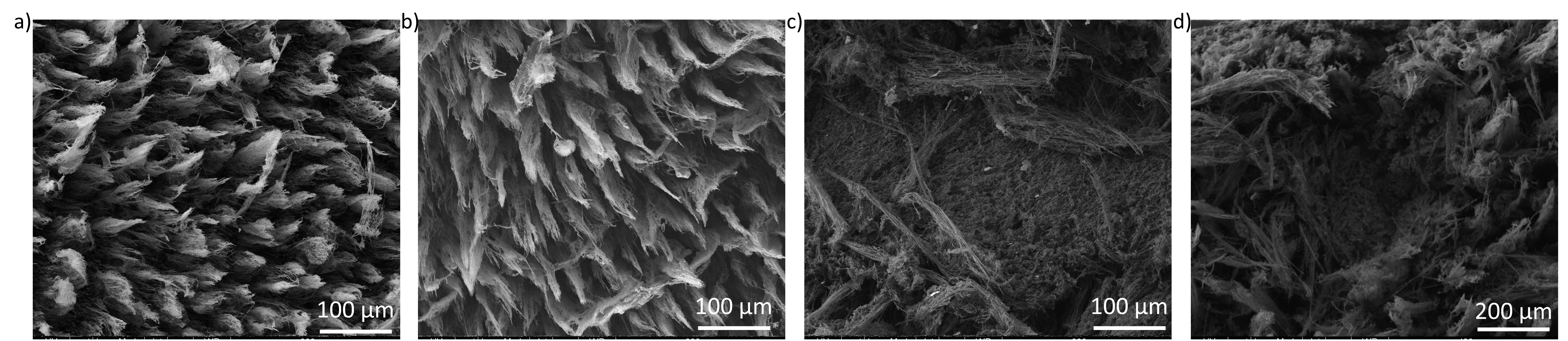


Figure S2: SEM images of squares engraved at a) 8 W, b) 12 W , c) 14 W and d) 16W


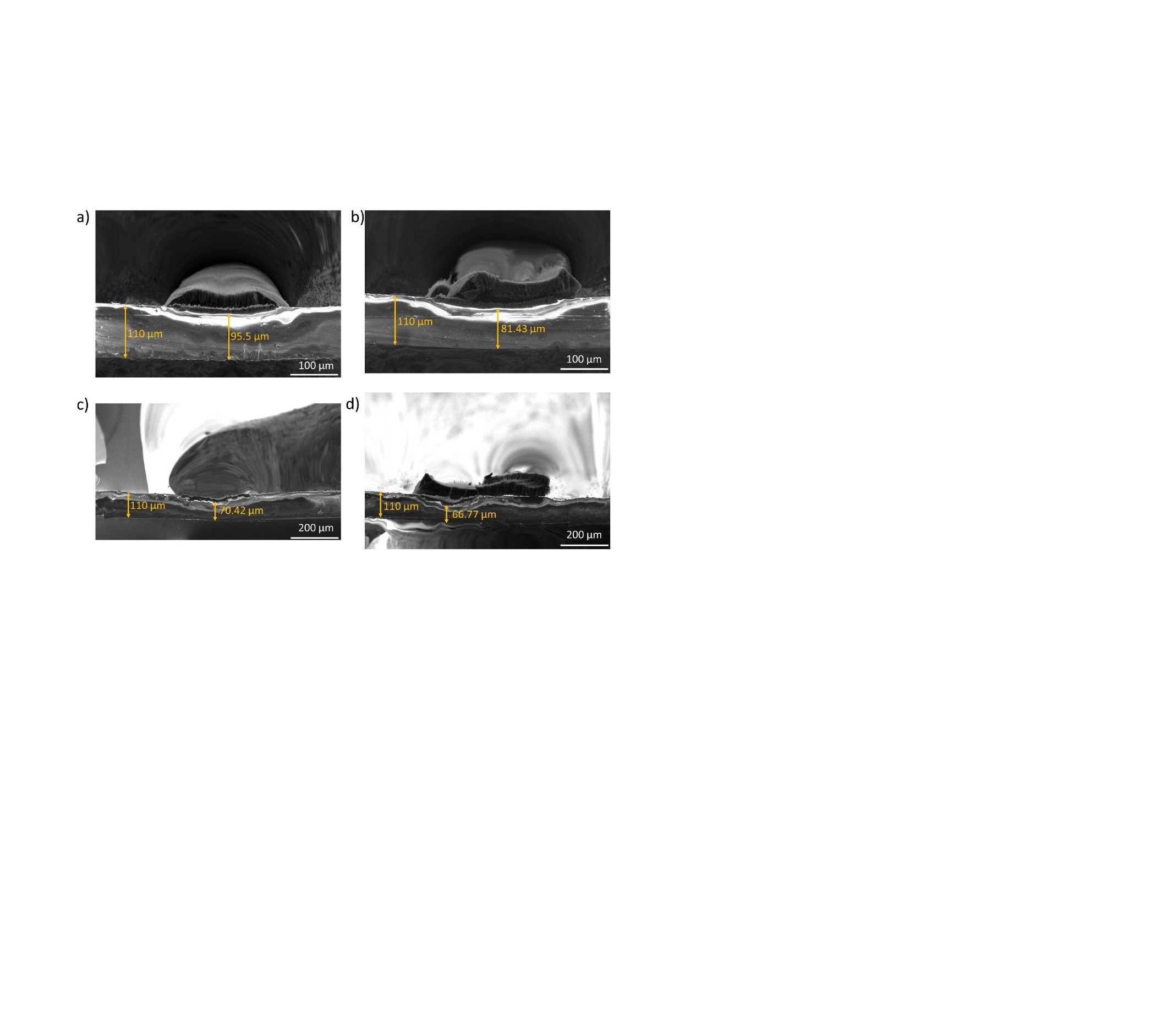


Figure S3: Cross sectional SEM images of engraved lines with calculated depth of engraving by image J software for a) 8W, b) 10 W, c) 14 W and d) 16 W


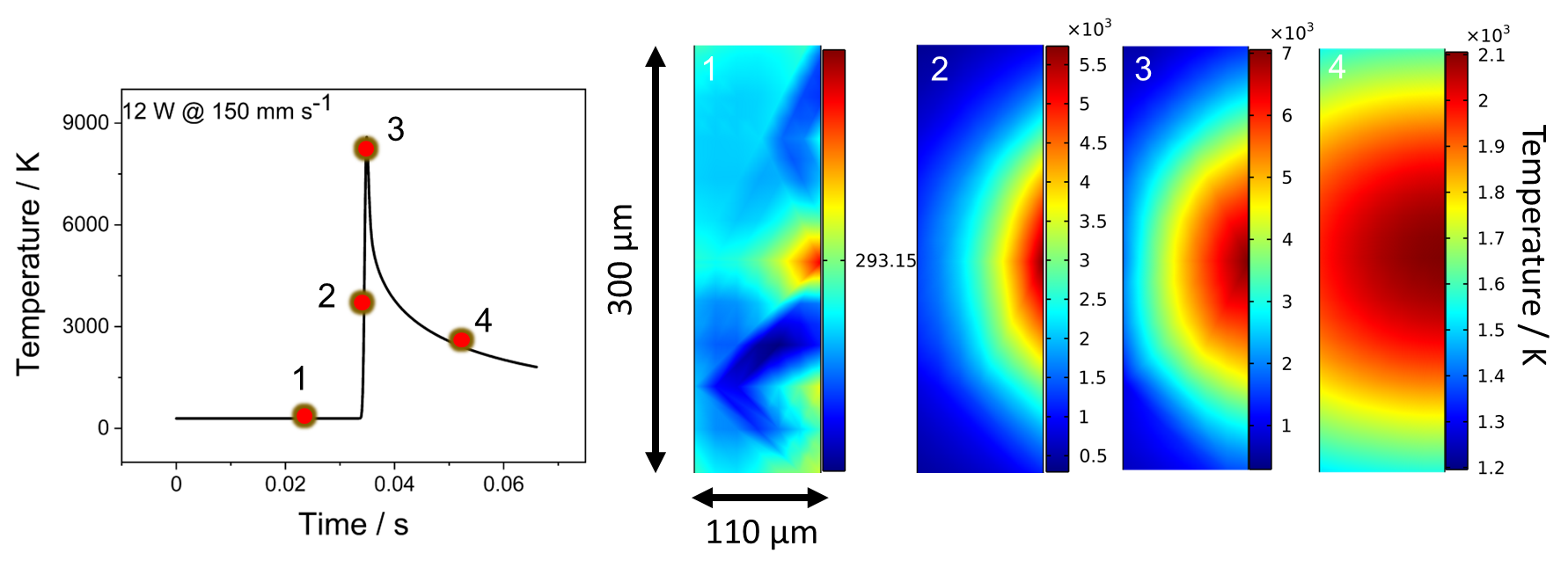


Figure S4: Plot of temperature versus the time for a particular scan rate evaluated for 12 W and corresponding temperature profile along the thickness


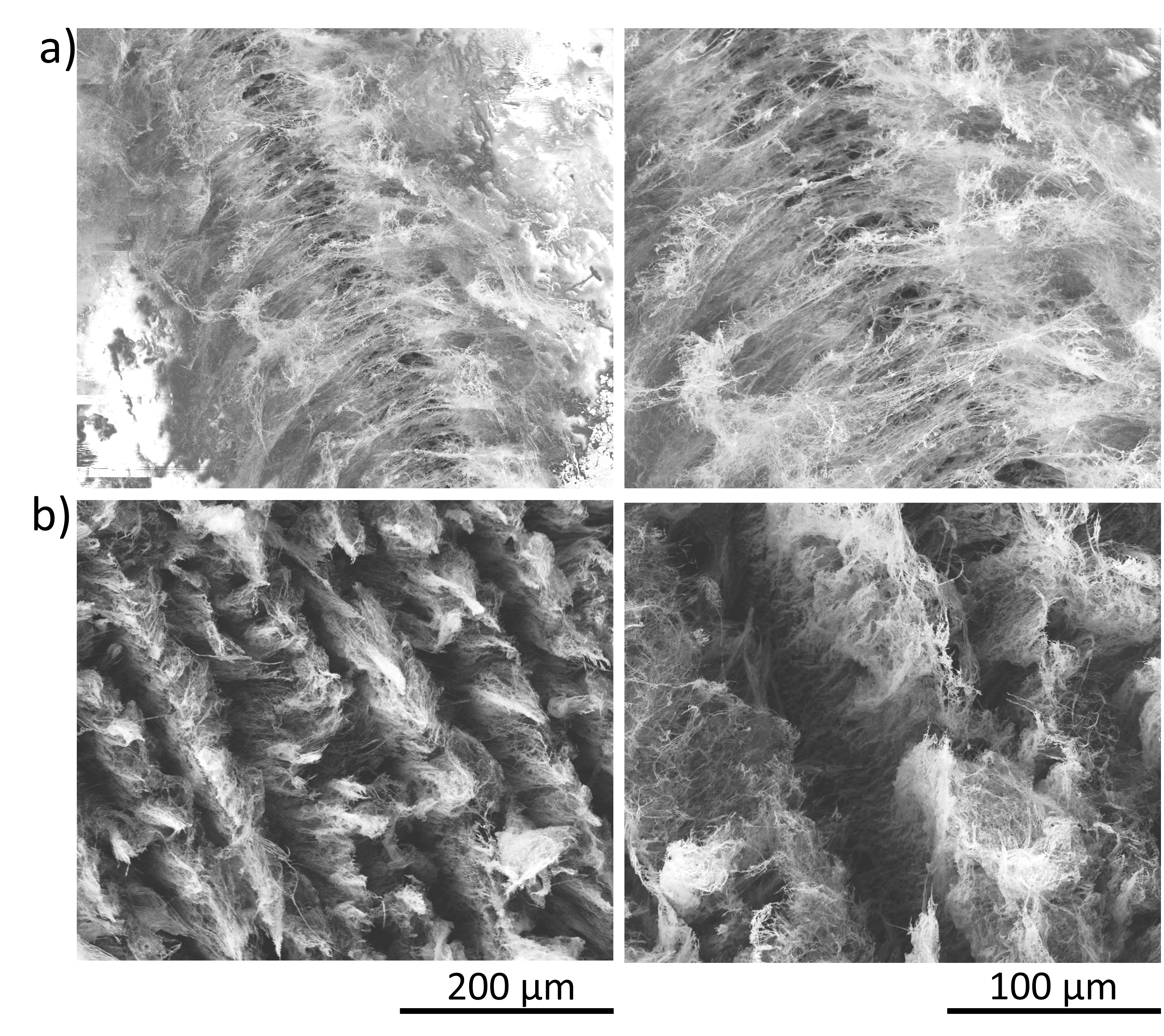


Figure S6: SEM images of lines engraved at 12 W and 150 mm s^-1^ with a) thickness of 0.025 mm and b) thickness of 1 mm
